# Physical activity, exercise habits and health-related quality of life in maintenance hemodialysis patients: a multicenter cross-sectional study

**DOI:** 10.1007/s40620-024-01935-6

**Published:** 2024-04-24

**Authors:** Huagang Hu, Pui Hing Chau, Edmond Pui Hang Choi

**Affiliations:** 1 School of Nursing, LKS Faculty of Medicine, The University of Hong Kong, 5/F, Academic Building, 3 Sassoon Road, Pokfulam, Hong Kong; 2 School of Nursing, Medical College, Soochow University, Box 203, 1 Shizi Street, Suzhou, 215006 Jiangsu Province China

**Keywords:** Maintenance hemodialysis, Physical activity, Exercise habits, Health-related quality of life

## Abstract

**Background:**

Although exercise has the potential to yield numerous benefits for maintenance hemodialysis patients, the relationship between physical activity, exercise habits, and health-related quality of life (HRQOL) has not been thoroughly investigated. This study aimed to characterize the physical activity, exercise habits, and HRQOL of maintenance hemodialysis patients while examining the associations between these factors.

**Methods:**

A convenience sampling was used to recruit 827 patients from 74 dialysis units in China. The structured questionnaire included sociodemographics, the International Physical Activity Questionnaire, exercise habits, and the Kidney Disease Quality of Life Short Form 1.3. An independent samples *t*-test, multivariable logistic regression analysis, and multivariable linear regression analysis were used.

**Results:**

The physical activity levels of 69% of participants were found to be below the recommendation of the World Health Organization. Despite 62.4% of participants reporting regular exercise, 73.8% reported light exercise intensity, and 92.6% engaged in walking or jogging. Participants’ HRQOL was higher in the low-intensity exercise group compared with the moderate-to-hard-intensity exercise group. Moderate-to-high physical activity was associated with better HRQOL in 13 of the 19 domains and regular exercise was linked to higher HRQOL in four of the 19 domains.

**Conclusions:**

This study identified a low level of physical activity, light exercise intensity, and walking as the primary exercise type among the participants. A significant correlation was found between higher physical activity levels, regular exercise, and better HRQOL for maintenance hemodialysis patients. It is advisable to implement strategies to enhance physical activity levels and design exercise programs for maintenance hemodialysis patients based on their real-world physical activity levels and exercise habits.

**Graphical abstract:**

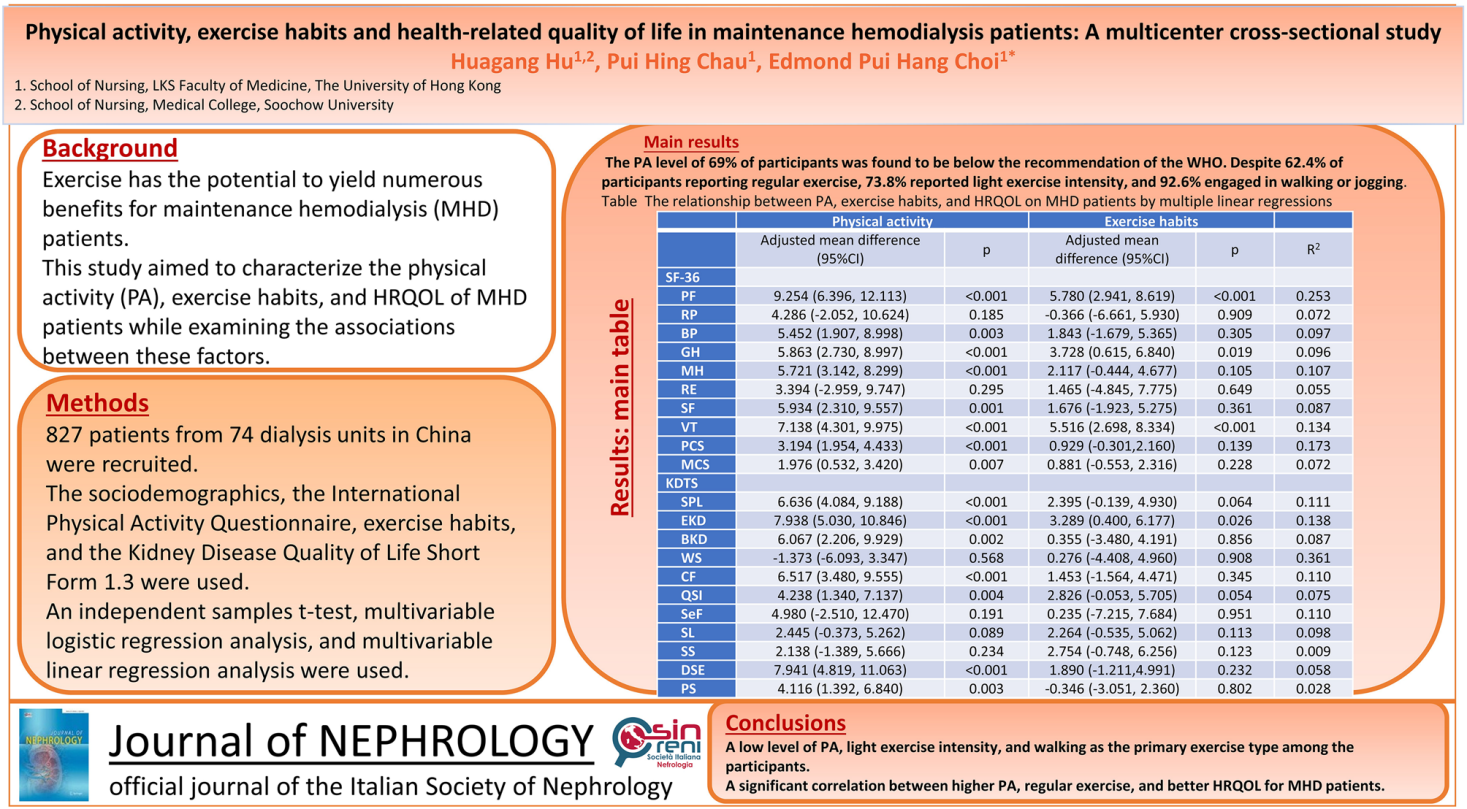

**Supplementary Information:**

The online version contains supplementary material available at 10.1007/s40620-024-01935-6.

## Introduction

Patients undergoing maintenance hemodialysis experience a range of symptom clusters and economic burdens, resulting in a lower health-related quality of life (HRQOL) compared to their healthy counterparts [1]. Physical activity refers to any body movements produced by the contraction of skeletal muscles [2]. Insufficient physical activity can result in adverse outcomes, such as poor HRQOL and increased mortality risk, for maintenance hemodialysis patients [1, 3–5]. However, physical activity levels tend to decline as kidney disease progresses [6, 7]. A cross-sectional study found that maintenance hemodialysis patients (*n* = 93) had significantly lower physical activity scores compared to healthy controls (*n* = 140), with higher physical activity scores correlating to better physical components summary of HRQOL [1]. A cohort study involving 317 maintenance hemodialysis patients reported that mortality risk decreased by 56% for each point increase in the Human Activity Profile maximal activity scores [5].

Exercise, a subset of physical activity, is characterized as a structured, planned, repetitive, and purposeful physical activity [2, 8]. A 6-month, multicenter randomized control trial with 227 dialysis patients showed that, compared with the usual care control group, a home-based personalized walking exercise program could improve the walking capacity and two domains of HRQOL, namely cognitive function domain and quality of social interaction domain [9]. Guidelines and published studies have reported that exercise could improve physical function and HRQOL for maintenance hemodialysis patients [10, 11]. Current evidence regarding exercise for maintenance hemodialysis patients mainly focuses on the effect of different types of exercises on outcomes such as HRQOL and physical fitness [11–14]. However, only a few studies explored the exercise habits of this population in a real -world setting [15]. An international study encompassing 12 countries and 20,920 dialysis patients assessed regular exercise through participants’ self-reports. The result indicated that only 47.4% of participants were classified as engaging in regular exercise. Nevertheless, the study combined the physical activity with exercise and only reported exercise frequency, which led to the omission of the other important components of exercise such as duration, type, and intensity [15].

Given the benefits of exercise that have been established through clinical trials, it is essential to comprehend the current state of exercise participation and its relationship with HRQOL in maintenance hemodialysis patients. In addition, it is advisable to design exercise programs for maintenance hemodialysis patients based on their real-world physical activity ability and exercise preference rather than just according to evidence from clinical trials. However, information on exercise habits in maintenance hemodialysis patients remains limited, particularly in China. Additionally, exercise and physical activity differ in definition and scope [2, 8], potentially leading to distinct relationships with HRQOL. Establishing a clear distinction between physical activity and exercise, as well as their connections with HRQOL, can assist healthcare providers in offering more specific advice to maintenance hemodialysis patients [8]. The relationships among physical activity, exercise habits, and HRQOL remain unclear for this population, therefore, this study aimed to describe the physical activity, exercise habits, and HRQOL of maintenance hemodialysis patients and explore the associations between them.

## Methods

### Study design and setting

This was a multicenter cross-sectional study. The Strengthening the Reporting of Observational Studies in Epidemiology (STROBE) statement was used to report this study. This survey included 74 dialysis units from 18 provinces in China. Patients undergoing maintenance hemodialysis were recruited between January and July 2022. The inclusion criteria were adulthood (age ≥ 18 years), having received maintenance hemodialysis for at least three months, and not experiencing any acute complications (e.g., acute infection, unstable angina and acute myocardial infarction). Patients who were too sick to participate or had been hospitalized within three months prior to the survey were excluded from the study.

The sample size was calculated using the formula: sample size = [Z_(1–α/2)_/d]^2^p(1–p) [16], where p represents the expected proportion of active maintenance hemodialysis patients. Referring to a published study, we assumed the expected proportion to be 6% [7] and we set the precision (*d*) at 0.02. With *α* = 0.05, *Z*_(1–α/2)_ = 1.96, resulting in a calculated sample size of 541. To account for a 20% incompletion rate, we planned to recruit at least 677 maintenance hemodialysis patients for the study.

### Data collection and measurements

In each dialysis unit, a trained investigator conducted the survey. The investigator recruited maintenance hemodialysis patients through interviews. After obtaining written consent, face-to-face interviews were carried out with the participants to complete the questionnaires.

The sociodemographic data included age, sex, height, body weight, body mass index, education, marital status, employment, smoking habits, and monthly income. Clinical characteristics, including dialysis vintages, hypertension, vascular access, type of blood purification, dialysis frequency, etiology of end-stage kidney disease, comorbidity, and dialysis efficacy, were extracted from the patients’ medical records. The Charlson comorbidity index (CCI) was used to assess the comorbidity of participants. Hospital characteristics of the dialysis units (i.e., hospital property and hospital level) are also included. In China, there are public hospitals and private hospitals. The hospital levels include the tertiary hospital, secondary hospital, and primary hospital.

The short version of the International Physical Activity Questionnaire (IPAQ) was used to assess the physical activity levels [17]. The IPAQ data were converted to metabolic equivalents of tasks (MET) for each activity, and then the categories of low, moderate, or high levels of physical activity were established according to the IPAQ scoring guidelines [17]. The test–retest reliability of the Chinese short IPQA was 0.84 [18]. We also reported whether the physical activity aligned with the World Health Organization (WHO) recommendation (aerobic exercise with moderate intensity physical activity ≥ 150 min a week or vigorous intensity physical activity ≥ 75 min a week) [19].

The exercise habits questionnaire was developed through a series of steps. First, an items pool was established based on the literature review of published studies [2, 12, 13, 19]. Second, an expert panel was formed, and two rounds of online panel meetings were conducted to discuss the preliminary questionnaire, ensuring its comprehensiveness in covering necessary items. Third, a pilot test for the exercise questionnaire was conducted with 11 maintenance hemodialysis patients. The exercise questionnaire was modified as needed in each step. Regular exercise was defined as structured, planned, repetitive (at least once per week), and purposeful physical activity with regular participation [2]. The test–retest reliability, assessed by intra-class correlation coefficient, was 0.91 and the content validity index was 0.97.

The Kidney Disease Quality of Life Short Form 1.3 (KDQOL-SF^1.3^) covers generic and kidney disease-targeted scale (KDTS) of HRQOL [20]. The generic part of HRQOL is the Medical Outcomes Study Short-Form (SF-36) [20]. The SF-36 consists of eight domains, which can be categorized into two components: the physical component summary and the mental component summary. The KDTS includes 43 items across 11 domains [20]. We used an Excel spreadsheet, as recommended by RAND [20], to score the KDQOL-SF^1.3^. The domains and component summary score of KDQOL-SF^1.3^ are shown as a standard score from 0 to 100, and a higher score means better HRQOL. The validity and reliability of the Chinese version of KDQOL-SF^1.3^ have been previously proven [21].

### Data analysis

First, categorical variables were expressed as numbers (*n*) with percentages (%), while continuous variables were expressed as means with standard deviations (SD). Second, sociodemographics and clinical characteristics between patients were compared using an independent *t*-test for continuous variables and an exact *χ*^*2*^ test for categorical variables. Third, multiple logistic regression analysis was used to explore the sociodemographics, hospital variables of the dialysis units, and clinical factors associated with physical activity levels and exercise habits. Adjusted odds ratio (*aOR*) with 95% confidence intervals (*95% CI*) were reported. Fourth, an exact *χ*^*2*^ test was used to compare the physical activity level between maintenance hemodialysis patients who did not exercise regularly and those who did. Fifth, an independent *t*-test was used to compare the HRQOL score between different patients. Further, *Cohen’s d* effect size was also calculated by calculating the mean difference between groups and then dividing the result by the pooled SD: small effect (*d* = 0.2), moderate effect (*d* = 0.5) and large effect (*d* = 0.8). Sixth, three linear regression models were employed to identify the relationship between physical activity, exercise habits, and HRQOL. The dependent variable was HRQOL, and the independent variables in the crude model were physical activity (with low physical activity as the reference group) and exercise habits (with lack of regular exercise as the reference group). To control the potential sociodemographics and disease-related confounders, multiple linear regressions (models 1 and 2) were conducted. These variables were selected based on published studies [1, 4, 22]. All variables were retained in the model regardless of their statistical significance, and the mean difference in terms of unstandardized coefficients with *95% CI* was reported. No data imputations were performed, and missing data were excluded from the respective analysis. The data were analyzed using SPSS version 28.0 (IBM Corporation, Armonk, NY, USA). The statistical significance level was set to 0.05.

## Results

### Sociodemographic characteristics

**Fig. 1:**
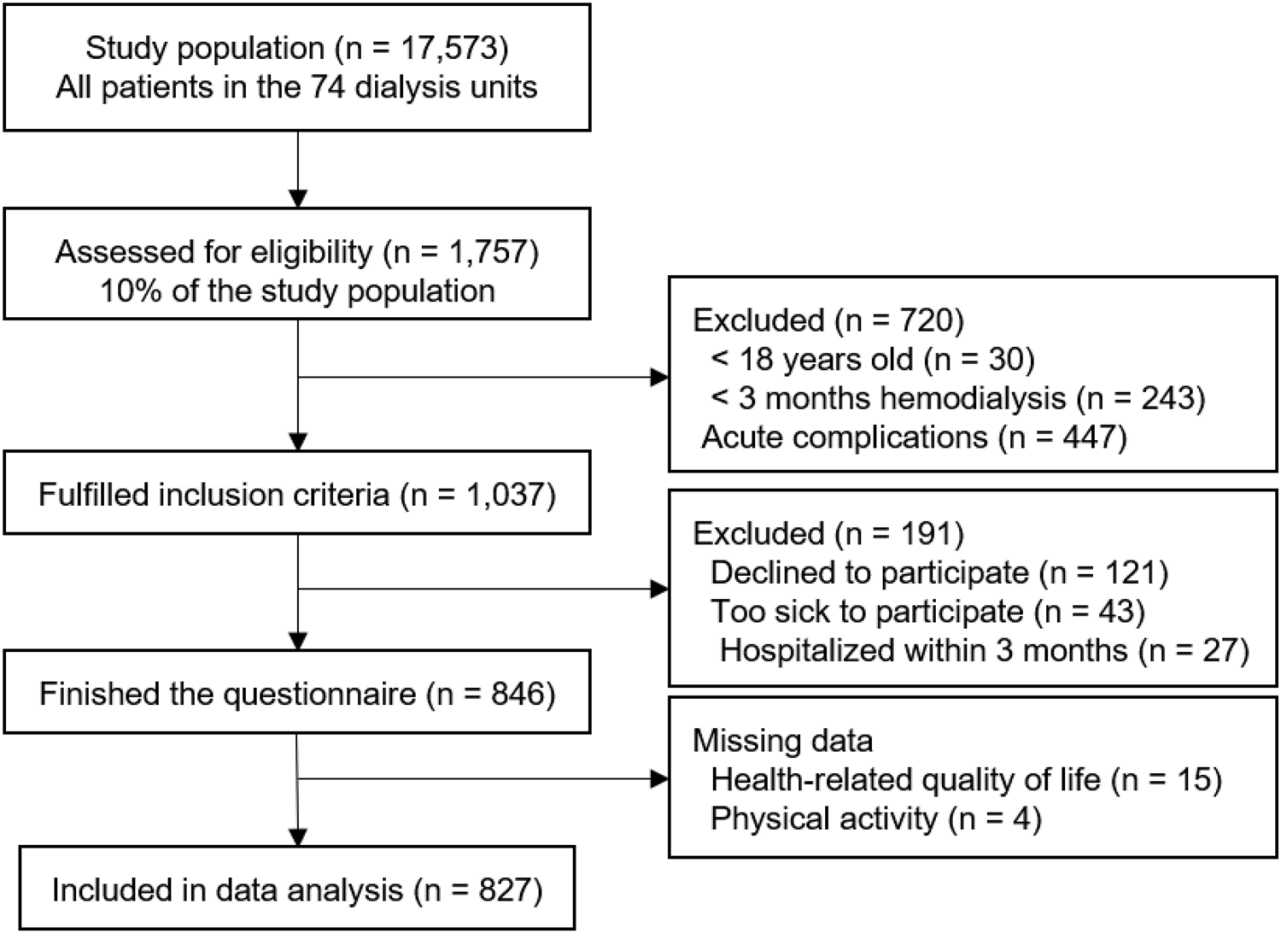
Flow chart for the cross-sectional study

A total of 827 maintenance hemodialysis patients, aged between 20 and 90 years, were included in the final data analysis (Fig. 1 and Table 1). The questionnaires missing the primary variables (HRQOL and physical activity) were excluded from the data analysis (Supplementary Table S1). These participants constituted approximately 5% of the total number of dialysis patients in the sampled dialysis unit. The majority of participants were male (61.9%), married (86.0%), and unemployed (60.9%).

**Table 1:** Characteristics of maintenance hemodialysis patients (*n* = 827)

Characteristics	*n* (%)	Characteristics	*n* (%)
Hospital property		Hypertension	
Public	727 (87.9)	No	251 (30.4)
Private	100 (12.1)	Yes	576 (69.6)
Hospital level		Vascular access	
Tertiary hospital	745 (90.1)	Arteriovenous fistula	763 (93.0)
Secondary hospital	82 (9.9)	Others ^a^	57 (7.0)
Sex		Dialysis vintage (months), mean (SD)	74.6 (68.2)
Male	512 (61.9)	Kt/V, mean (SD)	1.4 (0.4)
Female	315 (38.1)	Dialysis frequency	
Age (years), mean (SD)	48.6 (12.7)	Twice per week	62 (7.5)
Height (cm), mean (SD)	166.1 (7.8)	Thrice per week	765 (92.5)
Dry weight (kg), mean (SD)	61.7 (12.6)	Type of blood purification	
Body mass index (kg/m^2^), mean (SD)	22.2 (3.7)	Hemodialysis	250 (30.2)
Education level		Hemodialysis + hemodiafiltration/hemoperfusion	577 (69.8)
Below high school	348 (42.2)		
High school or above	477 (57.8)	Charlson comorbidity index	
Marital status		2–3	553 (67.2)
Unmarried	116 (14.0)	≥ 4	270 (32.8)
Married	710 (86.0)	Etiology of end-stage kidney disease	
Current employment		Glomerulonephritis	361 (44.2)
Yes	321 (39.1)	Hypertension	178 (21.8)
No	501 (60.9)	Diabetic nephropathy ^b^	76 (9.3)
Current smoker		Unknown	88 (10.8)
Yes	176 (21.3)	Congenital (birth) defects	49 (6.0)
No	651 (78.7)	IgA nephropathy	37 (4.5)
Monthly income		Tubulointerstitial disease	18 (2.2)
< 1000 CNY	86 (10.5)	Drug-induced nephritis	6 (0.7)
≥ 1000 CNY	733 (88.5)	Others	4 (0.5)

*CNY* China Yuan. ^a^ Arteriovenous graft, tunnel-cuffed catheter, or non-cuffed catheter; ^b^ Kidney biopsy- confirmed diabetic nephropathy with type 2 diabetes mellitus

### Physical activity, exercise habits, and HRQOL

More than one-third (36.3%) of the participants reported low physical activity and 69.0% of them had physical activity levels below the WHO’s recommended guidelines [19]. Among patients who reported regular exercise (62.4%), the majority (92.6%) engaged in walking or jogging, while only a small fraction (1.6%) performed resistance exercises (i.e., training with ankle weights, dumbbells, elastic bands, or fitness apparatus out of hemodialysis session). Most regular exercise participants reported exercising at least three times per week (86.9%), with light exercise intensity (73.8%), and not less than 20 min per exercise session (94.4%) (Table 2). For the generic aspect of HRQOL, the mean scores for physical function, bodily pain and mental health are greater than 70 (out of 100). For the KDTS aspect of HRQOL, the mean scores for dialysis staff encouragement, patient satisfaction, cognitive function, symptoms/problems list and social support are greater than 75 out of 100 (Table 2). Patients with at least a high school education and CCI scores below four were more likely to engage in moderate-to-high physical activity and with regular exercise (Supplementary Table S2). Factors associated with having moderate-to-high physical activity include having a high school or higher education and combining hemodialysis with hemodiafiltration/hemoperfusion. Similarly, factors associated with regular exercise habits included having a high school or above education, and height (Table 3). Patients who engage in regular exercise are more likely to report moderate-to-high physical activity compared to those who do not (Supplementary Table S3).

**Table 2:** Physical activity, exercise habits and health-related quality of life of maintenance hemodialysis patients (*n* = 827)

Variables	*n* (%)	Variables	Mean (SD)
**PA/Exercise**		**PA**	
Levels of PA		Total PA score ^a^	1890.6 (2347.6)
Low	300 (36.3)	Mild	941.3 (991.9)
Moderate	338 (40.9)	Moderate	580.7 (1084.7)
High	189 (22.8)	Vigorous	368.7 (1105.8)
Exercise regularly		PA as recommended by WHO ^b^, *n* (%)	
Yes	516 (62.4)	Yes	256 (31.0)
No	311 (37.6)	No	571 (69.0)
**Regular exercise**		**SF-36**	
Frequency of exercise		PF	74.3 (21.2)
< 3 per week	67 (13.1)	RP	53.6 (42.3)
≥ 3 per week	446 (86.9)	BP	73.7 (23.9)
Intensity of the exercise		GH	49.8 (21.1)
Light	380 (73.8)	MH	73.3 (17.5)
Moderate to hard	135 (26.2)	RE	64.7 (42.2)
Types of exercise		SF	68.5 (24.4)
Walking or jogging	478 (92.6)	VT	62.1 (19.5)
Tai Chi or Baduan Jin	21 (4.1)	PCS	41.5 (8.7)
Dancing or gymnastics	31 (6.0)	MCS	48.8 (9.7)
Climbing or climbing stairs	69 (13.4)	**KDTS**	
Cycling	53 (10.3)	SPL	77.9 (17.4)
Others aerobic exercise	19 (3.7)	EKD	63.8 (20.1)
Resistance exercise	8 (1.6)	BKD	39.8 (26.1)
Duration per session		WS	38.1 (37.9)
< 20 min	29 (5.6)	CF	78.1 (20.6)
≥ 20 min	485 (94.4)	QSI	74.1 (19.4)
When is the exercise done		SeF	72.9 (25.2)
Mainly on a non-dialysis day	295 (57.3)	SL	63.0 (19.3)
Mainly on dialysis day (out-of-dialysis)	34 (6.6)	SS	76.3 (22.8)
The same on dialysis and non-dialysis day	186 (36.1)	DSE	86.7 (20.8)
		PS	80.7 (17.9)

*BKD* burden of kidney disease, *BP* bodily pain, *CF* cognitive function, *DSE* dialysis staff encouragement, *EKD* effects of kidney disease, *GH* general health, *KDTS* kidney disease-targeted scales, *MCS* mental component summary, *MH* mental health, *PA* physical activity, *PCS* physical component summary, *PF* physical function, *PS* patient satisfaction, *QSI* quality of social interaction, *RE* role-emotional, *RP* role-physical, *SD* standard deviation, *SeF* sexual function, *SF* social function, *SF-36* Medical Outcomes Study Short-Form, *SL* sleep, *SPL* symptoms/problems list, *SS* social support, *VT* vitality, *WS* work status

^a^MET-minutes/week; ^b^Yes: moderate intensity PA ≥ 150 min per week; or vigorous intensity PA ≥ 75 min per week

**Table 3:** Factors associated with physical activity and exercise habits by multiple logistic regression

Independent variables	Physical activity^a^		Exercise habits^b^	
	*aOR (95%CI)*	*p*	*aOR (95%CI)*	*p*
Hospital property				
Public vs Private	0.633 (0.397, 1.011)	0.056	0.742 (0.464, 1.186)	0.212
Hospital level	1.092 (0.636, 1.876)	0.749		
Tertiary vs Secondary			1.150 (0.671, 1.969)	0.612
Sex				
Male vs Female	1.087 (0.701, 1.685)	0.709	1.190 (0.770, 1.840)	0.434
Age (years)	1.007 (0.995, 1.020)	0.264	1.011 (0.998, 1.024)	0.096
Height (cm)	0.992 (0.966, 1.019)	0.562	1.031 (1.004, 1.059)	0.023
Education level				
< High school vs ≥ High school	1.532 (1.101, 2.132)	0.011	1.895 (1.365, 2.631)	< 0.001
Monthly income				
< 1000 CNY vs ≥ 1000 CNY	0.961 (0.580, 1.592)	0.877	1.046 (0.635, 1.722)	0.860
Current smoker				
No vs Yes	0.978 (0.658, 1.455)	0.913	0.658 (0.445, 0.972)	0.035
Current employment				
Yes vs No	0.681 (0.481, 0.964)	0.030	0.987 (0.701, 1.389)	0.939
Vascular access				
Arteriovenous fistula vs Others	0.536 (0.297, 0.967)	0.038	0.763 (0.421, 1.385)	0.374
Dialysis frequency (per week)				
2 vs 3	1.618 (0.929, 2.820)	0.089	0.511 (0.272, 0.958)	0.036
Type of blood purification				
Hemodialysis vs Hemodialysis + Hemodiafiltration/Hemoperfusion	1.644 (1.179, 2.291)	0.003	1.361 (0.975, 1.900)	0.070
CCI				
2–3 vs ≥ 4	0.625 (0.453, 0.861)	0.004	0.688 (0.499, 0.948)	0.022

Physical activity, low vs moderate-to-high. Exercise habits, without regular exercise vs with regular exercise

*aOR* adjusted odds ratio, *CI* confidence interval, *CCI* Charlson comorbidityies index

^a^Model: chi-square = 48.529, df = 13, *p*-value < 0.001; Nagelkerke’s *R*-square = 0.082; Hosmer–Lemeshow Test, chi-square = 7.800, df = 8, *p*-value = 0.453

^b^Model: chi-square = 50.174, df = 13, *p*-value < 0.001; Nagelkerke’s *R*-square = 0.084; Hosmer–Lemeshow Test, chi-square = 8.809, df = 8, *p*-value = 0.359

Patients in the moderate-to-high physical activity group exhibited higher scores in all generic domains and most KDTS domains (9 out of 11) of HRQOL compared to the low physical activity group. Maintenance hemodialysis patients who exercised regularly had higher scores in the majority of generic domains (6 out of 8) and KDTS domains (8 out of 11) of HRQOL compared to those without regular exercise (Supplement Table S4). Patients with a higher exercise frequency, light intensity, at least 20 min duration per exercise session, and engaging in activities other than walking tended to have better HRQOL in certain domains (Supplementary Tables S5–S8).

### The relationship between physical activity, exercise habits and HRQOL

The relationship between physical activity, exercise habits, and HRQOL in maintenance hemodialysis patients is shown in Supplementary Tables 9, 10 (crude model and model 1) and Table 4 (model 2). After adjusting for sociodemographics, dialysis units, and clinical factors, moderate-to-high physical activity was significantly associated with higher scores in most generic domains (6 out of 8) and KDTS domains (7 out of 11) of HRQOL for maintenance hemodialysis patients. Regarding the generic aspect of HRQOL, the physical component summary for moderate-to-high physical activity was 3.194 (*95% CI* 1.954–4.433) units higher than low physical activity, while the mental component summary for moderate-to-high physical activity was 1.976 (*95% CI* 0.532–3.420) units higher than low physical activity (Table 4). After controlling for potential confounders, regular exercise was associated with higher scores in physical function, general health, vitality, and effects of kidney disease domains (Table 4).

**Table 4:** The relationship between physical activity, exercise habits and health-related quality of life on maintenance hemodialysis patients by multiple linear regressions (model 2)

	Physical activity		Exercise habits		
	Adjusted mean difference (95%CI)	*p*	Adjusted mean difference (95%CI)	*p*	*R*^2^
**SF-36**					
PF	9.254 (6.396, 12.113)	< 0.001	5.780 (2.941, 8.619)	< 0.001	0.253
RP	4.286 (− 2.052, 10.624)	0.185	− 0.366 (− 6.661, 5.930)	0.909	0.072
BP	5.452 (1.907, 8.998)	0.003	1.843 (− 1.679, 5.365)	0.305	0.097
GH	5.863 (2.730, 8.997)	< 0.001	3.728 (0.615, 6.840)	0.019	0.096
MH	5.721 (3.142, 8.299)	< 0.001	2.117 (− 0.444, 4.677)	0.105	0.107
RE	3.394 (− 2.959, 9.747)	0.295	1.465 (− 4.845, 7.775)	0.649	0.055
SF	5.934 (2.310, 9.557)	0.001	1.676 (− 1.923, 5.275)	0.361	0.087
VT	7.138 (4.301, 9.975)	< 0.001	5.516 (2.698, 8.334)	< 0.001	0.134
PCS	3.194 (1.954, 4.433)	< 0.001	0.929 (− 0.301, 2.160)	0.139	0.173
MCS	1.976 (0.532, 3.420)	0.007	0.881 (− 0.553, 2.316)	0.228	0.072
**KDTS**					
SPL	6.636 (4.084, 9.188)	< 0.001	2.395 (− 0.139, 4.930)	0.064	0.111
EKD	7.938 (5.030, 10.846)	< 0.001	3.289 (0.400, 6.177)	0.026	0.138
BKD	6.067 (2.206, 9.929)	0.002	0.355 (− 3.480, 4.191)	0.856	0.087
WS	− 1.373 (− 6.093, 3.347)	0.568	0.276 (− 4.408, 4.960)	0.908	0.361
CF	6.517 (3.480, 9.555)	< 0.001	1.453 (− 1.564, 4.471)	0.345	0.110
QSI	4.238 (1.340, 7.137)	0.004	2.826 (-0.053, 5.705)	0.054	0.075
SeF	4.980 (− 2.510, 12.470)	0.191	0.235 (− 7.215, 7.684)	0.951	0.110
SL	2.445 (− 0.373, 5.262)	0.089	2.264 (− 0.535, 5.062)	0.113	0.098
SS	2.138 (− 1.389, 5.666)	0.234	2.754 (− 0.748, 6.256)	0.123	0.009
DSE	7.941 (4.819, 11.063)	< 0.001	1.890 (− 1.211,4.991)	0.232	0.058
PS	4.116 (1.392, 6.840)	0.003	− 0.346 (− 3.051, 2.360)	0.802	0.028

*BKD* burden of kidney disease, *BP* bodily pain, *CF* cognitive function, *DSE* dialysis staff encouragement, *EKD* effects of kidney disease, *GH* general health, *KDTS* kidney disease-targeted scales, *MCS* mental component summary, *MH* mental health, *PA* physical activity, *PCS* physical component summary, *PF* physical function, *PS* patient satisfaction, *QSI* quality of social interaction, *RE* role-emotional, *RP* role-physical, *SeF* sexual function, *SF* social function, *SF-36* medical outcomes study short-form, *SL* sleep, *SPL* symptoms/problems list, *SS* social support, *VT* vitality, *WS* work status

The model is adjusted for hospital property, hospital level, sex, height, education level, work status, smoking, personal monthly income, type of blood purification, and Charlson comorbidity index

Physical activity, low vs moderate-to-high. Exercise habits, without regular exercise vs with regular exercise

## Discussion

This study found that maintenance hemodialysis patients exhibit low physical activity levels, light exercise intensity, and walking as their primary exercise type. Factors such as education, type of blood purification, and comorbidities were associated with physical activity levels and exercise habits. Interestingly, participants’ HRQOL was higher in the low-intensity exercise group compared with the moderate-to-hard-intensity exercise group. In addition, the study identified a significant positive association between physical activity levels (low vs moderate-to-high), exercise habits (no regular exercise vs regular exercise), and HRQOL in maintenance hemodialysis patients.

The WHO recommends that older adults with chronic conditions engage in at least 150 min of moderate-intensity physical activity or at least 75 min of vigorous-intensity physical activity per week [19]. It has been reported that 27.5% of adults do not meet these requirements [19], while 69.0% of participants in this study fell short. There are several reasons for the low physical activity levels among maintenance hemodialysis patients. First, they exhibit lower physical function than the general population, including decreased walking ability, cardiorespiratory function, and muscle strength [23]. Second, various barriers may prevent them from engaging in physical activity, such as fatigue, medical problems, lack of a suitable location, and inadequate social support [22, 24]. Third, regular hemodialysis is time-consuming, limiting the time available for physical activity in this population [22].

In our survey, 62.4% of maintenance hemodialysis patients reported regular exercise, which is higher than the 47.4% found in a published study [15]. Unlike the published research that defined regular exercise as engaging in exercise or physical activity at least once per week during leisure time [15], our study considered frequency, duration, type, and intensity of exercise and compared these characteristics with current guidelines [2, 19]. In our study, most maintenance hemodialysis patients (73.8%) who reported exercising regularly only participated in light-intensity activities, falling short of the recommendations by the WHO and the American College of Sports Medicine (ACSM) [2, 19]. The lower workload (time, duration, and intensity) of exercise may result from the poorer physical function of this population [13, 22, 25]. Our survey found that 92.6% of participants engaged solely in walking or jogging, with only 1.6% reporting resistance exercises, and none performing flexibility exercises as recommended by the ACSM [2]. This single exercise type may be due to the limited exercise knowledge and skills of maintenance hemodialysis patients [22, 25]. A published study indicated that a lack of information about exercise training was the main barrier for maintenance hemodialysis patients, who need support from their healthcare providers and family members [22]. However, nurses and nephrologists in hemodialysis units rarely provide exercise information, as they are less confident in their exercise knowledge and have heavy workloads [22, 24, 25]. Studies showed that exercise programs for maintenance hemodialysis patients could encourage more patients to participate in exercise and engage in more physical activity [26]. Nevertheless, only a limited number of dialysis units offer exercise training programs for maintenance hemodialysis patients [26, 27].

Patients who regularly exercise tend to have higher physical activity levels, but this does not necessarily mean they will achieve moderate-to-high levels of physical activity. Exercise is a subset of physical activity [8], so it is expected that individuals who exercise regularly will have higher physical activity levels compared to those who do not. As a broader concept than exercise, physical activity encompasses ambulation, daily living activities (e.g., housework, gardening, cleaning, etc.), work, and even leisure-time recreation [8, 28]. Thus, physical activity may primarily consist of the working environment, housework, and entertainment rather than regular exercise.

Maintenance hemodialysis patients with higher physical activity levels demonstrated better HRQOL in all generic domains and most KDTS domains (except for work status and sexual function). Maintenance hemodialysis patients who exercised regularly also experienced better HRQOL in most domains. These findings partially align with published studies suggesting that exercise can improve the physical component summary in maintenance hemodialysis patients [11]. Moreover, our study identified a positive relationship between moderate-to-high physical activity and the mental health of maintenance hemodialysis patients. The multiple linear regression analysis revealed that, after adjusting for potential confounders, moderate-to-high physical activity was associated with both the physical component summary and mental component summary, consistent with previously published studies [29]. Additionally, physical activity showed a greater association with the physical component (e.g., physical function) than the mental component (e.g., role-emotional) of HRQOL. This might be because more physical activity or exercise generally yields more physical benefits than mental ones [1, 11].

Regarding exercise habits, regular exercise was associated with four domains of HRQOL. These findings differ from past studies, which suggested that exercise could be beneficial for the physical component summary and most domains of HRQOL for the same population [11, 12]. In addition, a recent meta-analysis with 12 randomized clinical trials revealed that, home-based exercise could improve HRQOL in the physical role, emotional role, social functioning, bodily pain, and vitality domains as assessed by SF-36 among the dialysis population [30]. The discrepancy may arise from variations in study design (clinical trials vs cross-sectional survey) and exercise definitions (intradialytic exercise vs interdialytic exercise) [11, 12]. Therefore, further research is needed in real clinical scenarios to determine how to encourage exercise participation and improve HRQOL for maintenance hemodialysis patients.

This study identified the positive association between physical activity/exercise habits (e.g., higher physical activity levels, regular exercise, higher exercise frequency, and longer exercise duration) and HRQOL for maintenance hemodialysis patients, as recommended [2, 19]. However, it also revealed that HRQOL was higher in the low-intensity exercise group compared to the moderate-to-hard-intensity exercise group. This new finding prompts us to reconsider the recommendation of exercise intensity (moderate to hard, according to the WHO and ACSM) for this population [2, 19]. The results of this study may suggest that when designing exercise programs or making exercise recommendations for maintenance hemodialysis patients, in addition to referencing evidence from exercise guidelines and clinical trials (e.g., moderate to high exercise intensity), it is advisable to consider the real-world condition (e.g., light exercise intensity) to improve participation and compliance to exercise participation among the deconditioned population.

This study has some limitations. First, physical activity and exercise were assessed through questionnaires, which may introduce recall bias. However, the validity and credibility of the IPAQ were justified. Second, the convenience sampling methods used may limit the generalizability of this study. Nevertheless, the 827 participants in this study covered approximately 5% of the regular dialysis patients from 74 dialysis units across 18 provinces, making this study a good representation of this population in China. Third, the cross-sectional study design does not allow for establishing causality between physical activity, exercise habits, and HRQOL in this population.

## Conclusion

Patients undergoing maintenance hemodialysis report low physical activity levels, limited exercise participation, light exercise intensity, and walking as their primary exercise type. Higher physical activity levels and regular exercise are significantly associated with better HRQOL for maintenance hemodialysis patients. The findings of this study support the need for maintenance hemodialysis patients to engage in increased physical activity, regular exercise, and a variety of exercise types. Education regarding the benefits of physical activity/exercise and knowledge about exercise should be provided to maintenance hemodialysis patients. Considering that dialysis staff offer direct healthcare for maintenance hemodialysis patients, they play a critical role in improving the HRQOL of this population. Dialysis staff could assess patients’ physical activity levels and exercise abilities, and provide personalized advice to enhance their physical activity and exercise participation. It is advisable to implement strategies to enhance physical activity and design exercise programs for maintenance hemodialysis patients based on their real-world physical activity levels and exercise habits. Research on exercise among maintenance hemodialysis patients should not only focus on the effectiveness of the intervention but also consider the real-world implications of generalizing the intervention to the target population so that more patients can benefit.

## Supplementary Information

Below is the link to the electronic supplementary material.

Supplementary file1 (DOCX 79 KB)

## Data Availability

The datasets generated and/or analyzed during the current study are available from the corresponding author on reasonable request.
