# Supplementary material for: Physical activity, exercise habits and health-related quality of life in maintenance hemodialysis patients: a multicenter cross-sectional study
Source: J Nephrol. 2024 Apr 24;37(7):1881–91. doi: 10.1007/s40620-024-01935-6 (PMC11519245; doi:10.1007/s40620-024-01935-6)
Supplement: Supplementary file 1 — Supplementary file1 (DOCX 79 KB) [file 40620_2024_1935_MOESM1_ESM.docx]

**Table S1** Characteristics in participants included in and excluded from data analysis

| Characteristics | Included in data analysis  (n = 827) | Excluded from data analysis *  (n = 19) | *p* |
| --- | --- | --- | --- |
| Hospital property, n (%) |  |  | 0.836 |
| Public | 727 (87.9) | 17 (89.5) |  |
| Private | 100 (12.1) | 2 (10.5) |  |
| Hospital level, n (%) |  |  | 0.400 |
| Third level | 745 (90.1) | 16 (84.2) |  |
| Second level | 82 (9.9) | 3 (15.8) |  |
| Sex, n (%) |  |  | 0.912 |
| Male | 512 (61.9) | 12 (63.2) |  |
| Female | 315 (38.1) | 7 (36.8) |  |
| Age (years), mean (SD) | 48.58 (12.73) | 52.05 (14.92) | 0.242 |
| Height (cm), mean (SD) | 166.14 (7.81) | 166.25 (8.10) | 0.955 |
| Dry weight (kg), mean (SD) | 61.69 (12.63) | 58.42 (11.90) | 0.264 |
| BMI (kg/m^2^), mean (SD) | 22.24 (3.73) | 21.44 (3.70) | 0.394 |
| Education level, n (%) |  |  | 0.259 |
| Below high school | 348 (42.2) | 9 (56.3) |  |
| High school or above | 477 (57.8) | 7 (43.8) |  |
| Marital status, n (%) |  |  | 0.372 |
| Unmarried | 116 (14.0) | 1 (6.3) |  |
| Married | 710 (86.0) | 15 (93.8) |  |
| Current employment, n (%) |  |  | 0.420 |
| Yes | 321 (39.1) | 5 (29.4) |  |
| No | 501 (60.9) | 12 (70.6) |  |
| Current smoker, n (%) |  |  | 0.164 |
| Yes | 176 (21.3) | 6 (35.3) |  |
| No | 651 (78.7) | 11 (64.7) |  |
| Monthly income, n (%) |  |  | < 0.001 |
| < 1000 CNY | 86 (10.5) | 8 (50.0) |  |
| ≥ 1000 CNY | 733 (89.5) | 8 (50.0) |  |
| If hypertension, n (%) |  |  | 0.038 |
| No | 251 (30.4) | 10 (52.6) |  |
| Yes | 576 (69.6) | 9 (47.4) |  |
| Vascular access, n (%) |  |  | 0.547 |
| Arteriovenous fistula | 763 (93.0) | 17 (89.5) |  |
| Others | 57 (7.0) | 2 (10.5) |  |
| Dialysis vintage (months), mean (SD) | 74.58 (68.20) | 99.16 (64.54) | 0.120 |
| Kt/V, mean (SD) | 1.38 (0.35) | 1.38 (0.30) | 0.978 |
| Dialysis frequency, n (%) |  |  | 0.714 |
| Twice per week | 62 (7.5) | 1 (5.3) |  |
| Thrice per week | 765 (92.5) | 18 (94.7) |  |
| Type of blood purification, n (%) |  |  | 0.266 |
| Hemodialysis | 250 (30.2) | 8 (42.1) |  |
| Hemodialysis+  hemodiafiltration/heamoperfusion | 577 (69.8) | 11 (57.9) |  |
| Etiology of ESRD, n (%) |  |  | 0.964 |
| Glomerulonephritis | 361 (44.2) | 10 (52.6) |  |
| Hypertension | 178 (21.8) | 4 (21.1) |  |
| Diabetic nephropathy | 76 (9.3) | 1 (5.3) |  |
| Unknown | 88 (10.8) | 1 (5.3) |  |
| Congenital (birth) defects | 49 (6.0) | 2 (10.5) |  |
| IgA nephropathy | 37 (4.5) | 1 (5.3) |  |
| Tubulointerstitial disease | 18 (2.2) | 0 (0) |  |
| Drug-induced nephritis | 6 (0.7) | 0 (0) |  |
| Others | 4 (0.5) | 0 (0) |  |
| CCI, n (%) |  |  | 0.343 |
| 2-3 | 553 (67.2) | 14 (77.8) |  |
| ≥4 | 270 (32.8) | 4 (22.2) |  |

*BMI* body mass index, *CCI* Charlson comorbidities index, *ESRD* end-stage renal disease, *PA* physical activity

* Missing data: health-related quality of life (n = 15), physical activity (n = 4)

**Table S2** Physical activity and exercise habits in different maintenance hemodialysis patients

| Characteristics | Low PA  (n = 300) | Moderate-to-high PA (n = 527) | *p* | Without regular exercise  (n = 311) | With regular exercise  (n = 516) | *p* |
| --- | --- | --- | --- | --- | --- | --- |
| Hospital property, n (%) |  |  | 0.087 |  |  | 0.104 |
| Public | 256 (85.3) | 471 (89.4) |  | 266 (85.5) | 461 (89.3) |  |
| Private | 44 (14.7) | 56 (10.6) |  | 45 (14.5) | 55 (10.7) |  |
| Hospital level, n (%) |  |  | 0.506 |  |  | 0.841 |
| Third level | 273 (91.0) | 472 (89.6) |  | 281 (90.4) | 464 (89.9) |  |
| Second level | 27 (9.0) | 55 (10.4) |  | 30 (9.6) | 52 (10.1) |  |
| Sex, n (%) |  |  | 0.850 |  |  | 0.707 |
| Male | 187 (62.3) | 325 (61.7) |  | 190 (61.1) | 322 (62.4) |  |
| Female | 113 (37.7) | 202 (38.3) |  | 121 (38.9) | 194 (37.6) |  |
| Age (years), mean (SD) | 48.9 (14.0) | 48.4 (12.0) | 0.558 | 48.2 (13.5) | 48.8 (12.3) | 0.498 |
| Height (cm), mean (SD) | 166.3 (8.5) | 166.1 (7.4) | 0.735 | 165.4 (7.7) | 166.6 (7.9) | 0.033 |
| Dry weight (kg), mean (SD) | 61.8 (13.1) | 61.7 (12.4) | 0.917 | 61.4 (12.6) | 61.8 (12.7) | 0.667 |
| BMI (kg/m^2^), mean (SD) | 22.2 (44.0) | 22.3 (3.6) | 0.956 | 22.4 (3.9) | 22.2 (3.6) | 0.443 |
| Education level, n (%) |  |  | <0.001 |  |  | <0.001 |
| Below high school | 150 (50.2) | 198 (37.6) |  | 164 (52.9) | 184 (35.7) |  |
| High school or above | 149 (49.8) | 328 (62.4) |  | 146 (47.1) | 331 (64.3) |  |
| Marital status, n (%) |  |  | 0.657 |  |  | 0.191 |
| Unmarried | 40 (13.3) | 76 (14.4) |  | 50 (16.1) | 66 (12.8) |  |
| Married | 260 (86.7) | 450 (85.6) |  | 261 (83.9) | 449 (87.2) |  |
| Current employment, n (%) |  |  | 0.005 |  |  | 0.470 |
| Yes | 97 (32.7) | 224 (42.7) |  | 115 (37.5) | 206 (40.0) |  |
| No | 200 (67.3) | 301 (57.3) |  | 192 (62.5) | 309 (60.0) |  |
| Current smoker, n (%) |  |  | 0.703 |  |  | 0.025 |
| Yes | 66 (22.0) | 110 (20.9) |  | 79 (25.4) | 97 (18.8) |  |
| No | 234 (78.0) | 417 (79.1) |  | 232 (74.6) | 419 (81.2) |  |
| Monthly income, n (%) |  |  | 0.233 |  |  | 0.076 |
| < 1000 CNY | 36 (12.2) | 50 (9.5) |  | 40 (12.9) | 46 (9.0) |  |
| ≥ 1000 CNY | 259 (87.8) | 474 (90.5) |  | 269 (87.1) | 464 (91.0) |  |
| If hypertension, n (%) |  |  | 0.060 |  |  | 0.179 |
| No | 103 (34.3) | 148 (28.1) |  | 103 (33.1) | 148 (28.7) |  |
| Yes | 197 (65.7) | 379 (71.9) |  | 208 (66.9) | 368 (71.3) |  |
| Vascular access, n (%) |  |  | 0.007 |  |  | 0.475 |
| Arteriovenous fistula | 266 (89.9) | 497 (94.8) |  | 285 (92.2) | 478 (93.5) |  |
| Others | 30 (10.1) | 27 (5.2) |  | 24 (7.8) | 33 (6.5) |  |
| Dialysis vintage (months), mean (SD) | 73.4 (71.7) | 75.3 (66.2) | 0.695 | 71.8 (64.3) | 76.3 (70.5) | 0.363 |
| Kt/V, mean (SD) | 1.4 (0.3) | 1.4 (0.4) | 0.884 | 1.4 (0.3) | 1.4 (0.4) | 0.873 |
| Dialysis frequency, n (%) |  |  | 0.074 |  |  | 0.023 |
| Twice per week | 29 (9.7) | 33 (6.3) |  | 15 (4.8) | 47 (9.1) |  |
| Thrice per week | 271 (90.3) | 494 (93.7) |  | 296 (95.2) | 469 (90.9) |  |
| Type of blood purification, n (%) |  |  | <0.001 |  |  | 0.061 |
| Hemodialysis | 114 (38.0) | 136 (25.8) |  | 106 (34.1) | 144 (27.9) |  |
| Hemodialysis+  hemodiafiltration/heamoperfusion | 186 (62.0) | 391 (74.2) |  | 205 (65.9) | 372 (72.1) |  |
| Etiology of ESRD, n (%) |  |  | 0.341 |  |  | 0.861 |
| Glomerulonephritis | 139 (47.0) | 222 (42.6) |  | 129 (42.3) | 232 (45.3) |  |
| Hypertension | 55 (18.6) | 123 (23.6) |  | 67 (22.0) | 111 (21.7) |  |
| Diabetic nephropathy | 31 (10.5) | 45 (8.6) |  | 33 (10.8) | 43 (8.4) |  |
| Unknown | 26 (8.8) | 62 (11.9) |  | 30 (9.8) | 58 (11.3) |  |
| Congenital (birth) defects | 19 (6.4) | 30 (5.8) |  | 19 (6.2) | 30 (5.9) |  |
| IgA nephropathy | 12 (4.1) | 25 (4.8) |  | 14 (4.6) | 23 (4.5) |  |
| Tubulointerstitial disease | 10 (3.4) | 8 (1.5) |  | 9 (3.0) | 9 (1.8) |  |
| Drug-induced nephritis | 2 (0.7) | 4 (0.8) |  | 3 (1.0) | 3 (0.6) |  |
| Others | 2 (0.7) | 2 (0.4) |  | 1 (0.3) | 3 (0.6) |  |
| CCI, n (%) |  |  | <0.001 |  |  | 0.017 |
| 2-3 | 179 (59.9) | 374 (71.4) |  | 192 (62.1) | 361 (70.2) |  |
| ≥4 | 120 (40.1) | 150 (28.6) |  | 117 (37.9) | 153 (29.8) |  |

*BMI* body mass index, *CCI* Charlson comorbidities index, *ESRD* end-stage renal disease, *PA* physical activity

**Table S3** Physical activity in different exercise habits of maintenance hemodialysis patients, n (%)

|  | Without regular exercise  (n = 311) | With regular exercise  (n = 516) | *p* |
| --- | --- | --- | --- |
| Low physical activity | 173 (55.6) | 127 (24.6) | <0.001 |
| Moderate-to-high physical activity | 138 (44.4) | 389 (75.4) |  |

**Table S4** The health-related quality of life on maintenance hemodialysis patients with different physical activity levels and exercise habits (n = 827)

|  | Low PA,  Mean (SD) | Moderate-to-high PA,  Mean (SD) | *Cohen’s d* | *p* | Without regular exercise,  Mean (SD) | With regular exercise, Mean (SD) | *Cohen’s d* | *p* |
| --- | --- | --- | --- | --- | --- | --- | --- | --- |
| SF-36 |  |  |  |  |  |  |  |  |
| PF | 66.0 (25.4) | 79.0 (16.7) | 0.643 | < 0.001 | 68.2 (25.3) | 77.9 (17.4) | 0.468 | < 0.001 |
| RP | 49.1 (42.5) | 56.2 (42.1) | 0.169 | 0.020 | 51.0 (44.6) | 55.2 (40.9) | 0.101 | 0.160 |
| BP | 69.1 (25.3) | 76.4 (22.8) | 0.308 | < 0.001 | 70.7 (25.4) | 75.6 (22.8) | 0.206 | 0.004 |
| GH | 45.0 (19.7) | 52.6 (21.4) | 0.362 | < 0.001 | 46.1 (20.7) | 52.1 (21.1) | 0.288 | < 0.001 |
| MH | 68.5 (18.9) | 76.0 (16.0) | 0.440 | < 0.001 | 70.2 (19.0) | 75.2 (16.3) | 0.286 | < 0.001 |
| RE | 60.7 (43.3) | 67.0 (41.4) | 0.152 | 0.036 | 61.5 (44.1) | 66.7 (40.9) | 0.122 | 0.089 |
| SF | 63.6 (25.7) | 71.3 (23.2) | 0.320 | < 0.001 | 65.8 (26.5) | 70.2 (22.9) | 0.181 | 0.012 |
| VT | 56.1 (20.8) | 65.5 (17.9) | 0.499 | < 0.001 | 57.1 (20.9) | 65.1 (18.1) | 0.422 | < 0.001 |
| PCS | 39.0 (9.2) | 43.0 (8.1) | 0.476 | < 0.001 | 40.0 (9.5) | 42.4 (8.2) | 0.277 | < 0.001 |
| MCS | 47.0 (10.3) | 49.8 (9.2) | 0.294 | < 0.001 | 47.6 (10.2) | 49.6 (9.3) | 0.209 | 0.004 |
| KDTS |  |  |  |  |  |  |  |  |
| SPL | 72.8 (19.7) | 80.8 (15.2) | 0.474 | < 0.001 | 74.4 (18.9) | 80.0 (16.0) | 0.326 | < 0.001 |
| EKD | 57.5 (21.8) | 67.4 (18.1) | 0.508 | < 0.001 | 59.5 (22.1) | 66.4 (18.3) | 0.345 | < 0.001 |
| BKD | 34.5 (25.8) | 42.7 (25.8) | 0.318 | < 0.001 | 36.9 (26.2) | 41.5 (25.9) | 0.176 | 0.014 |
| WS | 35.2 (37.1) | 39.8 (38.3) | 0.121 | 0.097 | 36.3 (37.4) | 39.2 (38.2) | 0.077 | 0.287 |
| CF | 72.8 (22.3) | 81.1 (18.9) | 0.412 | < 0.001 | 75.0 (21.6) | 80.0 (19.7) | 0.248 | < 0.001 |
| QSI | 70.0 (21.3) | 76.5 (17.8) | 0.337 | < 0.001 | 71.0 (20.4) | 76.0 (18.5) | 0.261 | < 0.001 |
| SeF | 70.5 (28.8) | 73.9 (23.6) | 0.133 | 0.358 | 73.3 (27.6) | 72.7 (24.2) | 0.024 | 0.868 |
| SL | 59.9 (20.5) | 64.8 (18.3) | 0.255 | < 0.001 | 60.2 (20.4) | 64.7 (18.4) | 0.231 | 0.001 |
| SS | 74.0 (23.2) | 77.6 (22.5) | 0.156 | 0.032 | 73.6 (24.2) | 77.9 (21.7) | 0.189 | 0.009 |
| DSE | 80.7 (25.3) | 90.1 (16.9) | 0.457 | < 0.001 | 83.9 (22.8) | 88.3 (19.3) | 0.217 | 0.003 |
| PS | 78.2 (19.2) | 82.1 (17.0) | 0.221 | 0.002 | 80.3 (17.9) | 80.9 (17.9) | 0.035 | 0.623 |

*BKD* burden of kidney disease, *BP* bodily pain, *CF* cognitive function, *DSE* dialysis staff encouragement, *EKD* effects of kidney disease, *GH* general health, *KDTS* kidney disease-targeted scales, *MCS* mental component summary, *MH* mental health, *PA* physical activity, *PCS* physical component summary, *PF* physical function, *PS* patient satisfaction, *QSI* quality of social interaction, *RE* role-emotional, *RP* role-physical, *SD* standard deviation, *SeF* sexual function, *SF* social function, *SF-36* Medical Outcomes Study Short-Form, *SL* sleep, *SPL* symptoms/problems list, *SS* social support, *VT* vitality, *WS* work status

*Cohen’s d* effect size was determined by calculating the mean difference between groups and then dividing the result by the pooled SD.

**Table S5** The health-related quality of life of maintenance hemodialysis patients with different exercise frequency, Mean (SD)

|  | < 3 per week  (n = 67) | ≥ 3 per week  (n = 446) | *Cohen’s d* | *p* |
| --- | --- | --- | --- | --- |
| SF-36 |  |  |  |  |
| PF | 74.70 (18.19) | 78.42 (17.25) | 0.214 | 0.103 |
| RP | 42.54 (38.68) | 57.23 (40.91) | 0.362 | 0.006 |
| BP | 75.63 (21.96) | 75.68 (23.01) | 0.002 | 0.988 |
| GH | 48.66 (22.29) | 52.66 (20.92) | 0.190 | 0.149 |
| MH | 71.46 (15.08) | 75.69 (16.49) | 0.259 | 0.049 |
| RE | 57.71 (44.79) | 68.09 (40.11) | 0.255 | 0.052 |
| SF | 65.86 (21.82) | 70.94 (23.04) | 0.222 | 0.091 |
| VT | 62.24 (15.33) | 65.57 (18.45) | 0.184 | 0.160 |
| PCS | 40.69 (7.45) | 42.73 (8.24) | 0.251 | 0.056 |
| MCS | 47.66 (9.25) | 49.88 (9.26) | 0.240 | 0.067 |
| KDTS |  |  |  |  |
| SPL | 77.46 (14.93) | 80.38 (16.23) | 0.182 | 0.166 |
| EKD | 62.50 (18.23) | 66.89 (18.26) | 0.241 | 0.067 |
| BKD | 36.56 (24.36) | 42.17 (25.96) | 0.217 | 0.098 |
| WS | 40.00 (42.57) | 39.16 (37.69) | 0.022 | 0.870 |
| CF | 75.22 (19.72) | 80.72 (19.68) | 0.279 | 0.034 |
| QSI | 73.53 (18.42) | 76.32 (18.49) | 0.151 | 0.250 |
| SeF | 70.09 (19.94) | 73.27 (25.05) | 0.131 | 0.529 |
| SL | 65.11 (15.33) | 64.61 (18.83) | 0.027 | 0.837 |
| SS | 70.96 (21.94) | 78.96 (21.61) | 0.370 | 0.005 |
| DSE | 83.21 (24.96) | 89.07 (18.26) | 0.305 | 0.021 |
| PS | 78.86 (17.55) | 81.13 (18.00) | 0.127 | 0.334 |

*BKD* burden of kidney disease, *BP* bodily pain, *CF* cognitive function, *DSE* dialysis staff encouragement, *EKD* effects of kidney disease, *GH* general health, *KDTS* kidney disease-targeted scales, *MCS* mental component summary, *MH* mental health, *PCS* physical component summary, *PF* physical function, *PS* patient satisfaction, *QSI* quality of social interaction, *RE* role-emotional, *RP* role-physical, *SD* standard deviation, *SeF* sexual function, *SF* social function, *SF-36* Medical Outcomes Study Short-Form, *SL* sleep, *SPL* symptoms/problems list, *SS* social support, *VT* vitality, *WS* work status

*Cohen’s d effect size* was determined by calculating the mean difference between groups and then dividing the result by the pooled SD.

**Table S6** The health-related quality of life of maintenance hemodialysis patients with different exercise intensity, Mean (SD)

|  | Light  (n = 380) | Moderate-to-hard  (n = 135) | *Cohen’s d* | *p* |
| --- | --- | --- | --- | --- |
| SF-36 |  |  |  |  |
| PF | 79.00 (15.86) | 74.96 (20.87) | 0.233 | 0.020 |
| RP | 60.00 (39.98) | 42.22 (40.46) | 0.443 | <0.001 |
| BP | 78.34 (22.12) | 68.04 (23.20) | 0.460 | <0.001 |
| GH | 53.83 (20.84) | 47.52 (20.99) | 0.302 | 0.003 |
| MH | 76.78 (15.78) | 70.67 (17.04) | 0.379 | <0.001 |
| RE | 69.82 (39.22) | 58.27 (44.00) | 0.285 | 0.005 |
| SF | 72.40 (22.69) | 64.26 (22.56) | 0.359 | <0.001 |
| VT | 67.55 (17.35) | 58.41 (18.42) | 0.519 | <0.001 |
| PCS | 43.31 (7.76) | 40.04 (8.79) | 0.407 | <0.001 |
| MCS | 50.64 (8.91) | 46.71 (9.64) | 0.433 | <0.001 |
| KDTS |  |  |  |  |
| SPL | 81.84 (14.45) | 74.94 (19.05) | 0.437 | <0.001 |
| EKD | 68.21 (17.54) | 61.46 (19.29) | 0.375 | <0.001 |
| BKD | 42.80 (26.18) | 38.06 (24.51) | 0.184 | 0.067 |
| WS | 37.53 (37.77) | 43.99 (39.43) | 0.169 | 0.095 |
| CF | 82.50 (18.19) | 73.33 (22.02) | 0.476 | <0.001 |
| QSI | 77.30 (17.53) | 72.64 (20.48) | 0.254 | 0.012 |
| SeF | 73.14 (23.37) | 71.67 (26.44) | 0.061 | 0.728 |
| SL | 65.37 (18.35) | 62.74 (18.40) | 0.143 | 0.154 |
| SS | 77.84 (22.25) | 78.15 (20.41) | 0.014 | 0.887 |
| DSE | 89.61 (18.13) | 84.72 (21.95) | 0.255 | 0.011 |
| PS | 80.31 (18.45) | 82.47 (16.33) | 0.121 | 0.229 |

*BKD* burden of kidney disease, *BP* bodily pain, *CF* cognitive function, *DSE* dialysis staff encouragement, *EKD* effects of kidney disease, *GH* general health, *KDTS* kidney disease-targeted scales, *MCS* mental component summary, *MH* mental health, *PCS* physical component summary, *PF* physical function, *PS* patient satisfaction, *QSI* quality of social interaction, *RE* role-emotional, *RP* role-physical, *SD* standard deviation, *SeF* sexual function, *SF* social function, *SF-36* Medical Outcomes Study Short-Form, *SL* sleep, *SPL* symptoms/problems list, *SS* social support, *VT* vitality, *WS* work status

*Cohen’s d* effect size was determined by calculating the mean difference between groups and then dividing the result by the pooled SD.

**Table S7** The health-related quality of life of maintenance hemodialysis patients with different exercise duration, Mean (SD)

|  | < 20 minutes per session  (n = 29) | ≥ 20 minutes per session  (n = 485) | *Cohen’s d* | *p* |
| --- | --- | --- | --- | --- |
| SF-36 |  |  |  |  |
| PF | 67.41 (18.64) | 78.55 (17.13) | 0.647 | <0.001 |
| RP | 37.07 (36.97) | 56.55 (40.78) | 0.480 | 0.012 |
| BP | 63.97 (24.49) | 76.35 (22.58) | 0.546 | 0.004 |
| GH | 46.38 (17.21) | 52.47 (21.28) | 0.289 | 0.131 |
| MH | 70.90 (19.06) | 75.43 (16.15) | 0.278 | 0.147 |
| RE | 70.11 (39.18) | 66.67 (40.94) | 0.084 | 0.659 |
| SF | 57.33 (21.53) | 70.95 (22.80) | 0.599 | 0.002 |
| VT | 54.48 (17.75) | 65.78 (17.92) | 0.631 | 0.001 |
| PCS | 35.78 (5.93) | 42.85 (8.11) | 0.884 | <0.001 |
| MCS | 47.86 (10.62) | 49.71 (9.20) | 0.200 | 0.297 |
| KDTS |  |  |  |  |
| SPL | 77.37 (16.71) | 80.18 (16.04) | 0.175 | 0.360 |
| EKD | 59.81 (22.87) | 66.77 (17.93) | 0.382 | 0.046 |
| BKD | 30.17 (25.61) | 42.16 (25.78) | 0.465 | 0.015 |
| WS | 36.21 (35.09) | 39.48 (38.48) | 0.085 | 0.655 |
| CF | 74.02 (17.12) | 80.37 (19.84) | 0.322 | 0.092 |
| QSI | 73.33 (22.18) | 76.15 (18.27) | 0.152 | 0.426 |
| SeF | 70.83 (18.82) | 72.80 (24.46) | 0.081 | 0.846 |
| SL | 60.43 (15.02) | 64.98 (18.46) | 0.249 | 0.193 |
| SS | 77.59 (22.83) | 77.96 (21.74) | 0.017 | 0.928 |
| DSE | 82.33 (22.79) | 88.71 (19.06) | 0.331 | 0.084 |
| PS | 80.46 (18.40) | 80.96 (17.88) | 0.028 | 0.883 |

*BKD* burden of kidney disease, *BP* bodily pain, *CF* cognitive function, *DSE* dialysis staff encouragement, *EKD* effects of kidney disease, *GH* general health, *KDTS* kidney disease-targeted scales, *MCS* mental component summary, *MH* mental health, *PCS* physical component summary, *PF* physical function, *PS* patient satisfaction, *QSI* quality of social interaction, *RE* role-emotional, *RP* role-physical, *SD* standard deviation, *SeF* sexual function, *SF* social function, *SF-36* Medical Outcomes Study Short-Form, *SL* sleep, *SPL* symptoms/problems list, *SS* social support, *VT* vitality, *WS* work status

*Cohen’s d effect size* was determined by calculating the mean difference between groups and then dividing the result by the pooled SD.

**Table S8** The health-related quality of life of maintenance hemodialysis patients with different exercise types, Mean (SD)

|  | Walking or jogging  (n = 478) | Others  (n = 38) | *Cohen’s d* | *p* |
| --- | --- | --- | --- | --- |
| SF-36 |  |  |  |  |
| PF | 77.87 (17.24) | 78.95 (19.19) | 0.062 | 0.712 |
| RP | 54.92 (40.98) | 59.21 (39.60) | 0.105 | 0.533 |
| BP | 75.33 (22.82) | 78.88 (23.13) | 0.155 | 0.357 |
| GH | 52.14 (21.00) | 51.71 (22.25) | 0.021 | 0.903 |
| MH | 75.13 (16.12) | 75.47 (18.93) | 0.021 | 0.901 |
| RE | 66.04 (41.20) | 74.56 (35.88) | 0.209 | 0.216 |
| SF | 70.35 (23.09) | 68.42 (21.10) | 0.084 | 0.619 |
| VT | 65.17 (18.01) | 64.74 (18.89) | 0.024 | 0.888 |
| PCS | 42.37 (8.15) | 43.37 (8.28) | 0.123 | 0.467 |
| MCS | 49.61 (9.21) | 49.25 (10.24) | 0.039 | 0.818 |
| KDTS |  |  |  |  |
| SPL | 79.84 (16.26) | 82.29 (13.07) | 0.153 | 0.365 |
| EKD | 66.29 (18.35) | 67.52 (17.43) | 0.067 | 0.691 |
| BKD | 41.30 (25.99) | 43.59 (24.34) | 0.088 | 0.601 |
| WS | 38.79 (38.11) | 44.74 (39.91) | 0.155 | 0.357 |
| CF | 80.01 (19.63) | 80.18 (20.88) | 0.008 | 0.961 |
| QSI | 75.98 (18.31) | 76.49 (20.66) | 0.027 | 0.871 |
| SeF | 72.27 (24.74) | 77.50 (17.17) | 0.216 | 0.426 |
| SL | 64.44 (18.29) | 67.50 (19.33) | 0.167 | 0.323 |
| SS | 77.15 (21.97) | 87.28 (16.18) | 0.469 | 0.006 |
| DSE | 88.81 (19.08) | 82.57 (21.26) | 0.324 | 0.055 |
| PS | 81.14 (17.88) | 78.07 (18.63) | 0.171 | 0.311 |

*BKD* burden of kidney disease, *BP* bodily pain, *CF* cognitive function, *DSE* dialysis staff encouragement, *EKD* effects of kidney disease, *GH* general health, *KDTS* kidney disease-targeted scales, *MCS* mental component summary, *MH* mental health, *PCS* physical component summary, *PF* physical function, *PS* patient satisfaction, *QSI* quality of social interaction, *RE* role-emotional, *RP* role-physical, *SD* standard deviation, *SeF* sexual function, *SF* social function, *SF-36* Medical Outcomes Study Short-Form, *SL* sleep, *SPL* symptoms/problems list, *SS* social support, *VT* vitality, *WS* work status

*Cohen’s d effect size* was determined by calculating the mean difference between groups and then dividing the result by the pooled SD.

**Table S9** The relationship between physical activity，exercise habits and health-related quality of life of maintenance hemodialysis patients by multiple linear regressions (crude model)

|  |  | Physical activity | | Exercise habits | | |
| --- | --- | --- | --- | --- | --- | --- |
|  | *R^2^* | *Mean difference* (95%CI) | *P* | *Mean difference* (95%CI) | *P* |  |
| SF-36 |  |  |  |  |  |  |
| PF | 0.104 | 11.077 (8.076, 14.079) | <0.001 | 6.263 (3.283, 9.242) | <0.001 |  |
| RP | 0.005 | 6.414 (0.102, 12.725) | 0.046 | 2.279 (-3.986, 8.543) | 0.475 |  |
| BP | 0.022 | 6.377 (2.839, 9.914) | <0.001 | 2.943 (-0.568, 6.454) | 0.100 |  |
| GH | 0.035 | 6.258 (3.157, 9.360) | <0.001 | 4.094 (1.016, 7.172) | 0.009 |  |
| MH | 0.046 | 6.626 (4.069, 9.182) | <0.001 | 2.914 (0.376, 5.451) | 0.024 |  |
| RE | 0.004 | 5.275 (-1.012, 11.561) | 0.100 | 3.509 (-2.731, 9.748) | 0.270 |  |
| SF | 0.023 | 7.020 (3.413, 10.627) | <0.001 | 2.230(-1.350, 5.811) | 0.222 |  |
| VT | 0.070 | 7.700 (4.883, 10.516) | <0.001 | 5.690 (2.894, 8.485) | <0.001 |  |
| PCS | 0.052 | 3.663 (2.390, 4.936) | <0.001 | 1.266 (0.003, 2.530) | 0.049 |  |
| MCS | 0.021 | 2.428 (0.995, 3.861) | <0.001 | 1.266 (-0.157, 2.688) | 0.081 |  |
| KDTS |  |  |  |  |  |  |
| SPL | 0.055 | 6.945 (4.422, 9.468) | <0.001 | 3.441 (0.936, 5.945) | 0.007 |  |
| EKD | 0.063 | 8.599 (5.694, 11.503) | <0.001 | 4.179 (1.297, 7.062) | 0.005 |  |
| BKD | 0.022 | 7.501 (3.648, 11.354) | <0.001 | 2.249 (-1.575, 6.074) | 0.249 |  |
| WS | 0.001 | 4.064 (-1.634, 9.763) | 0.162 | 1.674 (-3.973, 7.322) | 0.561 |  |
| CF | 0.039 | 7.452 (4.439, 10.466) | <0.001 | 2.756 (-0.235, 5.747) | 0.071 |  |
| QSI | 0.030 | 5.393 (2.543, 8.244) | <0.001 | 3.351 (0.522, 6.180) | 0.020 |  |
| SeF | 0.004 | 3.830 (-3.659, 11.319) | 0.315 | -1.679 (-9.135, 5.777) | 0.658 |  |
| SL | 0.018 | 3.864 (1.011, 6.717) | 0.008 | 3.225 (0.393, 6.056) | 0.026 |  |
| SS | 0.008 | 2.433 (-0.960, 5.826) | 0.160 | 3.544 (0.177, 6.912) | 0.039 |  |
| DSE | 0.045 | 8.706 (5.668, 11.743) | <0.001 | 1.819 (-1.195, 4.834) | 0.237 |  |
| PS | 0.009 | 4.138 (1.475, 6.801) | 0.002 | -0.651 (-3.295, 1.992) | 0.629 |  |

Physical activity, low vs moderate to high; Exercise, without regular exercise vs with regular exercise

*BKD* burden of kidney disease, *BP* bodily pain, *CF* cognitive function, *DSE* dialysis staff encouragement, *EKD* effects of kidney disease, *GH* general health, *KDTS* kidney disease-targeted scales, *MCS* mental component summary, *MH* mental health, *PCS* physical component summary, *PF* physical function, *PS* patient satisfaction, *QSI* quality of social interaction, *RE* role-emotional, *RP* role-physical, *SD* standard deviation, *SeF* sexual function, *SF* social function, *SF-36* Medical Outcomes Study Short-Form, *SL* sleep, *SPL* symptoms/problems list, *SS* social support, *VT* vitality, *WS* work status

**Table S10** The relationship between physical activity, exercise habits and health-related quality of life of maintenance hemodialysis patients by multiple linear regressions (model 1)

|  |  | Physical activity | | | Regular exercise | | |
| --- | --- | --- | --- | --- | --- | --- | --- |
|  | *R^2^* | *Mean difference* (95%CI) | *P* | *Mean difference* (95%CI) | | *P* |  |
| SF-36 |  |  |  |  | |  |  |
| PF | 0.195 | 10.658 (7.727, 13.588) | <0.001 | 6.389 (3.454, 9.323) | | <0.001 |  |
| RP | 0.033 | 5.990 (-0.399, 12.379) | 0.066 | 0.677 (-5.719, 7.074) | | 0.835 |  |
| BP | 0.055 | 6.474 (2.892, 10.056) | <0.001 | 2.435 (-1.151, 6.021) | | 0.183 |  |
| GH | 0.053 | 6.645 (3.479, 9.811) | <0.001 | 4.209 (1.040, 7.379) | | 0.009 |  |
| MH | 0.052 | 6.680 (4.057, 9.302) | <0.001 | 2.579 (-0.046, 5.205) | | 0.054 |  |
| RE | 0.032 | 5.415 (-0.940, 11.770) | 0.095 | 2.383 (-3.980, 8.745) | | 0.462 |  |
| SF | 0.041 | 7.421 (3.751, 11.090) | <0.001 | 2.238 (-1.436, 5.911) | | 0.232 |  |
| VT | 0.090 | 8.155 (5.282, 11.027) | <0.001 | 5.954 (3.078, 8.830) | | <0.001 |  |
| PCS | 0.125 | 3.620 (2.359, 4.880) | <0.001 | 1.176 (-0.086, 2.438) | | 0.068 |  |
| MCS | 0.032 | 2.541 (1.082, 4.000) | <0.001 | 1.123 (-0.338, 2.583) | | 0.132 |  |
| KDTS |  |  |  |  | |  |  |
| SPL | 0.066 | 7.314 (4.732, 9.895) | <0.001 | 2.812 (0.227, 5.397) | | 0.033 |  |
| EKD | 0.082 | 8.951 (5.987, 11.915) | <0.001 | 3.807 (0.840, 6.774) | | 0.012 |  |
| BKD | 0.072 | 6.955 (3.115, 10.796) | <0.001 | 0.791 (-3.054, 4.636) | | 0.687 |  |
| WS | 0.362 | -1.176 (-5.841, 3.490) | 0.621 | 0.472 (-4.189, 5.134) | | 0.842 |  |
| CF | 0.058 | 7.593 (4.509, 10.677) | <0.001 | 2.126 (-0.962, 5.213) | | 0.177 |  |
| QSI | 0.041 | 5.294 (2.380, 8.208) | <0.001 | 3.318 (0.401, 6.236) | | 0.026 |  |
| SeF | 0.106 | 4.874 (-2.568, 12.316) | 0.198 | -0.030 (-7.420, 7.360) | | 0.994 |  |
| SL | 0.044 | 3.657 (0.776, 6.538) | 0.013 | 2.995 (0.111, 5.879) | | 0.042 |  |
| SS | 0.002 | 2.646 (-0.848, 6.141) | 0.138 | 3.027(-0.470, 6.524) | | 0.090 |  |
| DSE | 0.044 | 8.583 (5.476, 11.689) | <0.001 | 2.214(-0.896, 5.324) | | 0.163 |  |
| PS | 0.027 | 4.466 (1.776, 7.155) | 0.001 | -0.248 (-2.941, 2.444) | | 0.856 |  |

This model is adjusted for age, sex, high, education level, work status, smoking, personal monthly income.

Physical activity, low vs moderate to high; Exercise, without regular exercise vs with regular exercise.

*BKD* burden of kidney disease, *BP* bodily pain, *CF* cognitive function, *DSE* dialysis staff encouragement, *EKD* effects of kidney disease, *GH* general health, *KDTS* kidney disease-targeted scales, *MCS* mental component summary, *MH* mental health, *PCS* physical component summary, *PF* physical function, *PS* patient satisfaction, *QSI* quality of social interaction, *RE* role-emotional, *RP* role-physical, *SD* standard deviation, *SeF* sexual function, *SF* social function, *SF-36* Medical Outcomes Study Short-Form, *SL* sleep, *SPL* symptoms/problems list, *SS* social support, *VT* vitality, *WS* work status
